# Efficient and cost-effective non-invasive population monitoring as a method to assess the genetic diversity of the last remaining population of Amur leopard (*Panthera pardus orientalis*) in the Russia Far East

**DOI:** 10.1371/journal.pone.0270217

**Published:** 2022-07-06

**Authors:** Sujoo Cho, Puneet Pandey, Jee Yun Hyun, Taisia Marchenkova, Anna Vitkalova, Timophey Petrov, Daecheol Jeong, Jangmi Lee, Dong Youn Kim, Ying Li, Yury Darman, Mi-Sook Min, Kyung Seok Kim, Victor Bardyuk, Hang Lee

**Affiliations:** 1 Research Institute for Veterinary Science and Conservation Genome Resource Bank for Korean Wildlife, College of Veterinary Medicine, Seoul National University, Seoul, South Korea; 2 Tiger and Leopard Conservation Fund in Korea, Seoul, South Korea; 3 ENPROTEC India Foundation, Uttar Pradesh, India; 4 Federal State Budgetary Institution Joint Directorate of Kedrovaya Pad’ State Biosphere Nature Reserve and Land of the Leopard National Park, Ministry of Natural Resources and Environment of the Russian Federation, Vladivostok, Primorsky Krai, Russia; 5 College of Geography and Ocean Science, Yanbian University, Yanji, Jilin, China; 6 WWF-Russia, Amur Branch, Vladivostok, Primorsky Krai, Russia; 7 Department of Natural Resource Ecology and Management, Iowa State University, Ames, Iowa, United States of America; Zoological Survey of India, INDIA

## Abstract

Small populations of the endangered species are more vulnerable to extinction and hence require periodic genetic monitoring to establish and revisit the conservation strategies. The Amur leopard is critically endangered with about 100 individuals in the wild. In this study, we developed a simple and cost-effective noninvasive genetic monitoring protocol for Amur leopards. Also, we investigated the impact of fecal sample’s age, storage, and collection season on microsatellite genotyping success and data quality. We identified 89 leopard scats out of the 342 fecal samples collected from Land of the Leopard between 2014–2019. Microsatellite genotyping using 12 markers optimized in 3 multiplex PCR reactions reveals presence of at least 24 leopard individuals (18 males and 6 females). There was a significant difference in the success rate of genotyping depending on the time from feces deposition to collection (*p* = 0.014, Fisher’s exact test), with better genotyping success for samples having <2 weeks of environmental exposure. Amur leopard genetic diversity was found low (H_o_− 0.33, H_E_− 0.35, and N_A_− 2.57) with no visible population substructure and recent bottleneck signature. Although a historical bottleneck footprint was observed. Mitochondrial DNA diversity was also found low with two haplotypes differing by a point mutation reported in 1,769 bp of investigated sequence covering parts of cytochrome *b* gene (846 bp), *NADH-5* gene (611 bp) and control region (312 bp). We recommend periodic genetic monitoring of wild Amur leopards following the proposed methodology to achieve cost effectiveness and efficiency.

## Introduction

Increased anthropogenic disturbances, such as land development, over exploitation, pollution, introduction of exotic species, are threatening many wildlife around the world [1]. In the past few centuries, several species went extinct and many more on the verge of extinction due to habitat loss/fragmentation and reduction in population size to unsustainable levels [2–4]. Small populations are especially vulnerable to extinction due to environmental and demographic factors in addition to inbreeding [5–8]. Therefore, continued population monitoring to understand the species’ population trend is critical to establish a conservation strategy for species remaining in a very small number [9, 10].

The Amur leopard (*Panthera pardus orientalis* Schlegel, 1857), the northernmost leopard subspecies, is critically endangered [11, 12] and has severely low genetic diversity [13]. Historically, it was distributed over a wide range of East Asia including the Korean Peninsula, northeastern China, and the southern part of the Russian Far East [14, 15]. A rapid population decline was observed in the 20^th^ century mainly due to excessive hunting, declining of prey species and marginalization of habitat resources, and only a small population currently exists in the mountain-forest region near the borders of Russia, China, and North Korea [12, 13, 16, 17]. According to snow tracking surveys conducted in Russia from the 1990s to early 2000s, the number of leopards has been retained at 20–40 individuals in the southwestern part of Primorsky Krai [18–20]. In 2012, the Land of the Leopard National Park, Primorsky Krai, Russia Far East was established to conserve and restore the last surviving Amur leopard population. Since then, the population has continued to gradually recover to approximately 100 individuals currently inhabiting the protected area (leopard-land.ru) [21].

In this study, we developed a simple and cost-effective microsatellite based DNA fingerprinting protocol to identify leopard individuals using the DNA extracted from feces. Further, the genetic diversity of critically endangered Amur leopards was analyzed noninvasively (fecal DNA) by monitoring microsatellite markers and mitochondrial DNA (mtDNA). Additionally, factors (i.e., sample age, storage, and month of collection) influencing the performance of noninvasive genetic studies were assessed. The study is part of an ongoing Korea–Russia collaborative wildlife research project to develop long–term population management plan for critically endangered Amur leopard and Amur tiger (*Panthera tigris altaica* Temminck, 1844).

## Material and methods

### Sampling and DNA extraction

**Fig 1:**
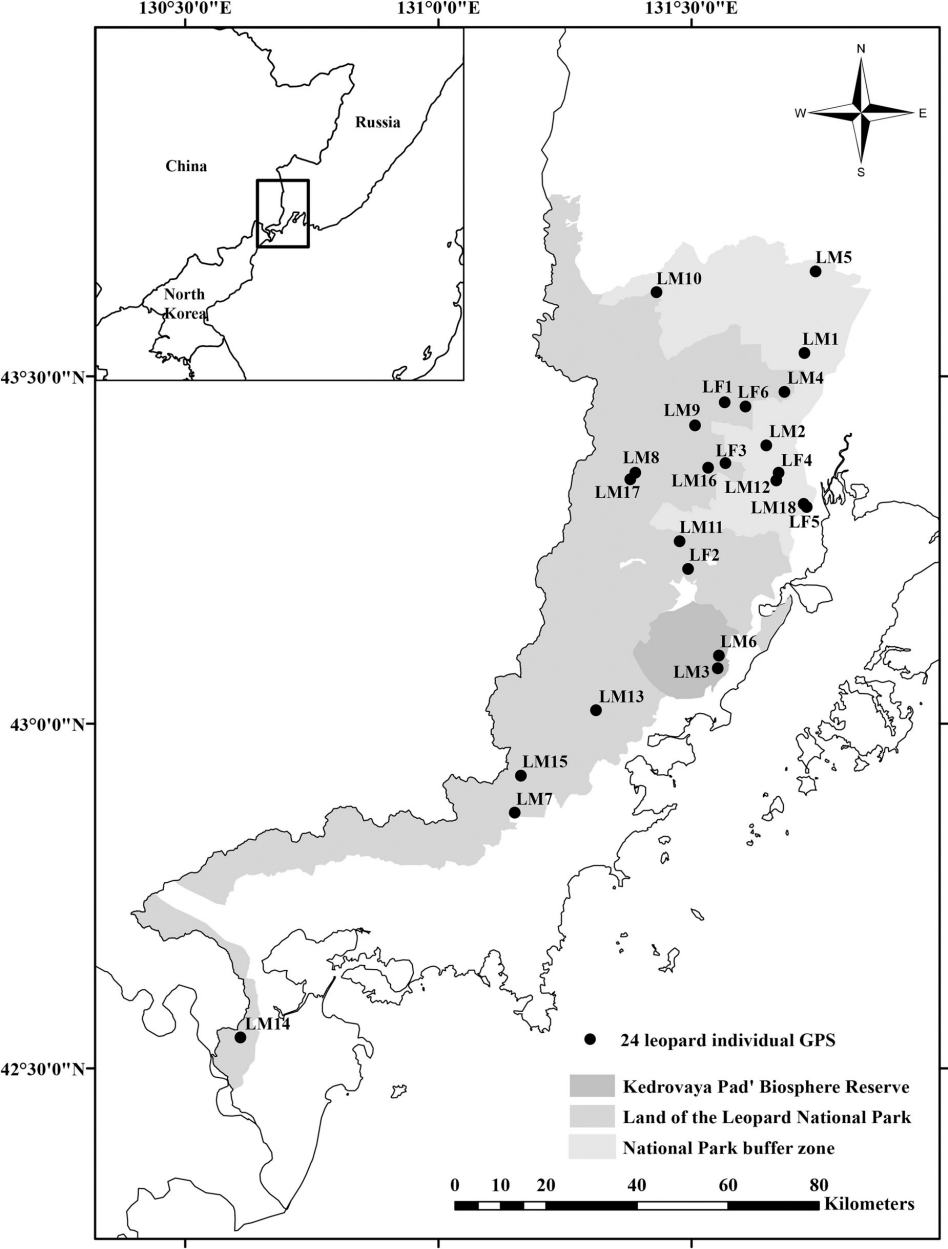
Capture location of individual Amur leopards identified by a genetic survey. The study site (Land of the Leopard) is shown in gray. The representative sample GPS locations of 24 individuals are depicted as black dots.

Fecal samples (n = 342) were collected by park rangers and researchers opportunistically from the Land of the Leopard (Fig 1) during six winter tracking surveys conducted from 2014 to 2019. Land of the Leopard is collectively referred to the Land of the Leopard National Park, National Park buffer zone and the Kedrovaya Pad’ Biosphere Reserve (totally 3690 km^2^). Precautions were enacted during sample collection to avoid sample contamination and the collection date, Global Positioning System (GPS) coordinates, approximate age of the sample, type of collection site, and presumed species were recorded. Scat sample age at the time of collection was determined by experienced field biologists based on scat morphology (intact or disintegrated), appearance (colour) and moisture content (moist or dry). Also, nearest surrounding area for traces and scrapes were investigated to determine the sample age. Each sample was assigned a unique identification number, and stored in plastic bags at −20°C in a freezer prior to genomic DNA (gDNA) extraction, which was conducted using the QIAamp Fast DNA Stool Mini Kit (Qiagen, Hilden, Germany) in accordance with the manufacturer’s protocol. Negative controls were included during DNA extraction to monitor contamination and success. DNA extraction was conducted in Russia and the DNA extracts were exported to South Korea for genetic analysis (CITES permit no. ES2019-03989). All samples were legally and ethically collected following noninvasive sampling approaches. The study involved no capture or handling of any live animal. The study protocol was conducted in accordance with the ethical guidelines of the Institutional Animal Care and Use Committee of Seoul National University (Seoul, South Korea).

### Species and sex identification

Land of the Leopard hosts both tigers and leopards. During sample collection, the collected samples were assigned to a specific species by field biologists and re-verified by multiplex polymerase chain reaction (PCR) and agarose gel electrophoresis as described by Sugimoto et al. [22]. Multiplex PCR was conducted with primer sets, specific to the Amur tiger and Amur leopard. Species identity was ascertained based on differences in amplicon size by agarose gel electrophoresis (271 vs. 156 bp for the tiger and leopard, respectively). Samples were examined up to four times until a species-specific band was clearly detected. Then, 10 leopard and tiger samples were randomly selected for revalidation of the species identification results by sequencing of 308 bp of the cytochrome *b* region using the universal primers L14841 and H15149 [23], followed by alignment with sequences retrieved from the National Center for Biotechnology Information (NCBI) database (https://www.ncbi.nlm.nih.gov/) using the Basic Local Alignment Search Tool (https://blast.ncbi.nlm.nih.gov/Blast.cgi).

The gender to fecal sample was determined based on amplification of genes located on X chromosome (ZFX) and Y chromosome (DBY-7) [22]. The fragment sizes of ZFX and DBY-7 were 205 and 156 bp, respectively. The primers used were specific to tiger and leopard and do not amplify DNA of the prey species. At least three independent assays were conducted and consistent results of two of the assays was required to assign sex to a sample. The multiplex PCR mixture and PCR cycling condition was same as described in Sugimoto et al. [22] for species and sex identification. Negative and positive controls were included in the assay.

### Microsatellite genotyping

DNA fingerprinting based on microsatellite genotyping of the fecal samples was used to identify unique individuals and subsequently estimate the genetic diversity of the Amur leopard population. The whole process included following three steps: (i) microsatellite prescreening (selection of loci for individual identification), (ii) identification of unique individuals by genotyping of fecal samples with select markers, and (iii) estimation of the genetic diversity of the Amur leopard population. Microsatellite markers developed for big cat species were used to identify individuals and assess genetic diversity [24]. In total, 35 randomly selected leopard fecal samples were genotyped using 32 microsatellite markers specific to big cats with eight multiplex PCR reactions (MPP1–8) [24]. Four loci were amplified in each multiplex PCR reaction. Monomorphic markers that cannot distinguish different individuals or calculate genetic diversity were excluded. Polymorphic markers associated with a high probability of identity of unrelated individuals (P_ID_) and lower genotyping errors (allele dropout and false allele) were selected in order to use the minimum number of markers with sufficient discrimination power. The use of fewer markers can lower the probability of errors and increase efficiency. The remaining leopard samples were amplified by the selected marker and individual identification for the entire leopard samples proceeded.

Individual identification was performed using samples with more than 70% of the loci successfully genotyped. Genotyping errors inevitably occur with the use of fecal DNA extracts because degraded DNA remains in small amounts with inhibitors [25, 26]. Thus, changes to the number of identified individuals with the number of allowed mismatches (genotyping error) was assessed and the appropriate number of mismatches was determined by spotting a point where the number of individuals did not significantly change. Samples were grouped to have as many similar alleles as possible. A maximum of three mismatched loci was allowed to prevent population overestimation caused by genotyping errors [27, 28]. The number of identified individuals was stable between 2 and 3 mismatches, indicating allowing three mismatches is suitable. The excluded polymorphic markers were additionally used to reconfirm the individual identification results and genetic diversity was calculated.

The microsatellite loci were amplified by PCR in 10 μL reaction volumes consisting of 5 μL of Qiagen multiplex kit master mix, 0.5 μL of Q-Solution, 0.25 μL of each primer (10 pmol), and 2.0 μL of DNA extract. The PCR conditions for amplification of microsatellites included an initial denaturation step at 94°C for 15min followed by 45 cycles of denaturation at 94°C for 40 s, annealing at 60°C for MPP1–7 or 55°C for MPP8 for 1 min, and extension at 72°C for 1min, and a final extension step at 72°C for 30 min. Each round of PCR included a positive and negative control to validate the PCR procedure and detect contamination. The PCR products were analyzed using ABI 3730xl genetic analyzer (Applied Biosystems, Foster City, CA, USA) at NICEM Inc. (Seoul, South Korea), and the genotyping data were collected using Geneious Prime 2020.1.2 (https://www.geneious.com).

Consensus genotypes of samples were determined by threshold method (consensus threshold = 3) of GIMLET, which was also used to calculate the allelic dropout and false allele rates [29]. The presence of null alleles and genotyping errors due to scoring or stuttering was checked in the Micro-Checker 2.2.3 [30]. Linkage disequilibrium between all pairs of polymorphic loci and the Hardy-Weinberg equilibrium of each locus were assessed using the GENEPOP following Bonferroni correction [31]. Basic genetic diversity parameters such as observed and expected allele richness (A, and A_E_) and gene diversity (H_O_, and H_E_), and marker’s polymorphic information content (PIC) were estimated using GenAlEx 6.5 [32] and CERVUS [33]. The P_ID_ and probability of identity among siblings (P_IDsib_) were calculated using GIMLET [29].

Since the Amur leopard population has experienced a significant decline in the past, the presence of a contemporary and historical bottleneck signature was investigated. BOTTLENECK 1.2.02 was used to investigate the presence of a contemporary bottleneck with 1000 iterations at two-phase mutational model (variance, 30%; stepwise mutation model, 70%) [34]. The Wilcoxon signed rank test was employed as a statistically robust test that provides reliable results with fewer samples and markers as compared to other tests. To check for the presence of historical bottleneck, the Garza-Williamson index (M-ratio) [35] was calculated using ARLEQUIN 3.5.2.2 [36].

### mtDNA sequence analysis

Three segments of mtDNA (total of 1,769 bp) were amplified, which included parts of the cytochrome *b* gene (846 bp), *NADH-5* gene (611 bp) and control region (312 bp). The cytochrome *b* gene of the Amur leopard was amplified with the primer sets PSF1/PSR1 and PSF2/PH1 as described by Sugimoto et al. [37], and *NADH-5* gene was amplified with the primer sets F/RL2 and FL2/RL4 as described by Uphyrkina et al. [38]. To amplify the part of control region hypervariable segment-1 adjoining the 5′ end of the central conserved region, the forward primer PC1 and reverse primer PC2 were designed using Primer3 [39] embedded in the Geneious Prime 2020.1.2 (https://www.geneious.com) (S1 Table). PC2 was modified from the H16498 primer of Shields et al. [40] to match the *Panthera pardus* sequence. The PCR products were gel extracted and sequenced by Macrogen Inc. (Seoul, South Korea) on an ABI 3730xl genetic analyzer (Applied Biosystems, Foster City, CA, USA). The sequences were analyzed using Geneious Prime 2020.1.2 (https://www.geneious.com) and the peak quality of each sequence was checked manually. Segments were aligned to the published whole mitochondrion sequence of *P*. *p*. *orientalis* (KX655614) and to the comparable partial sequences of *P*. *p*. *orientalis* (cytochrome *b*: AB817078 and AB817079; *NADH-5*: AY035260, AY035261, and MK114159; control region: AY035227, AY035228, and MK114160). The emergence and loss of unique genetic variants were also investigated.

### Sample quality and genotyping success

Rangers recorded the sample collection date and estimated the age of fecal sample based on the scat morphology, appearance and moisture content. During analysis, the time from deposition to collection was categorized as <2, 2–4, or >4 weeks, while the time from collection to DNA extraction was categorized as <2 years, 2–4 years, and >4 years, and collection month were categorized as November–December, January, February, and March–April. Samples without information were not categorized. To investigate whether the age of sample, storage duration, or the month of collection were correlated with the success rate of genotyping, the Fisher’s exact test was conducted for three separate cases between sample quality and (1) time from deposition to collection, (2) time from collection to DNA extraction and (3) month of collection. The samples were classified as good, medium, or poor quality based on the amplification success (consensus genotype) of the 12 microsatellite loci selected for individual identification (good, 9–12 loci amplified; medium, 5–8 loci amplified; poor, 0–4 loci amplified, S2 Table).

## Results

### Species and sex determination

Of the 342 fecal samples, 89 (4 in 2013–2014, 15 in 2014–2015, 12 in 2015–2016, 11 in 2016–2017, 16 in 2017–2018, 30 in 2018–2019, 1 unknown) were from leopards (S3 Table). Of the remaining 253 fecal samples, 242 were of tiger and 11 could not be amplified or identified. Sex was determined for 64 out of 89 leopard samples (male, n = 57; female, n = 7). The greater number of fecal samples collected from male Amur leopards is likely due to opportunistic sample collection as reported in previous noninvasive genetic studies of Amur leopards and Amur tigers [37, 41]. The success rate of leopard and tiger species identification was 97% and leopard sex determination was 72%. Sequencing of the partial fragment of the cytochrome *b* gene followed by NCBI blast revalidated the PCR-gel based species identification protocol and resultantly all tested samples (n = 10) assigned to the same species. We further compared field biologist observations about species of origin of each of the collected samples and results of genetic experiment. Of the 342 scats, field biologists assigned species of origin to 309 scats. Genetic species identification results were found similar to field observation for 284 samples (92%). The remaining samples include 2 Tiger (genetically identified as leopard) and 14 Leopard (genetically identified as tiger). Nine samples were not amplified or genetically identified as different species although field observation was thought to be tiger or leopard.

### Genotyping and marker selection

Of the 32 screened microsatellite markers, 17 were monomorphic and 15 were polymorphic (S4 Table). Of the 15 polymorphic markers, 12 were selected for identification of individual Amur leopards based on the number of alleles and probability of identity value of 35 randomly selected samples (S4 Table). Three polymorphic markers were not used when identifying individuals, because two markers (Pan5D1 and Pan2C1) had low discriminating power and one marker (Pan6A1) had a high error rate and difficulty of allele scoring. Although Pan6A1 was excluded, Pan5D1 and Pan2C1 were used for further analysis (genetic diversity assessment and bottleneck test). Combinations of the 12 markers for individual identification are listed in Table 1. In total, 51 samples (3 in 2013–2014, 8 in 2014–2015, 6 in 2015–2016, 5 in 2016–2017, 10 in 2017–2018, 18 in 2018–2019, 1 unknown) were successfully genotyped at least 9 of 12 loci and used for individual identification. The resultant probability of identity of 12 markers for unrelated individuals and siblings were 1.32 × 10^−5^ and 7.16 × 10^−3^, respectively, which were deemed sufficient to distinguish among the estimated 100 individuals inhabiting the Land of the Leopard [21, 42] (leopard-land.ru). Inclusion of Pan5D1 and Pan2C1 had no effect on the individual identification results. When limited to 14 loci, the allelic dropout rate per locus was 0–0.222 and the false allele rate was 0–0.093. The average allelic dropout and false allele rates were 0.068 and 0.033, respectively (Table 2). There was no significant deviation from the Hardy–Weinberg equilibrium and no strong evidence of linkage disequilibrium between any pair of loci. In addition, there was no evidence of null alleles.

**Table 1:** Panel of the microsatellite markers proposed for Amur leopard population monitoring.

Multiplex PCR	Marker	Dye Label	Annealing temperature (°C)	Success* (%)
1	Pan3C2	6FAM	60	87
	Pan4A2	VIC		69
	Pan2A1	NED		60
	Pan16C2	PET		63
2	Pan5A1	6FAM	60	60
	Pan14C2	VIC		31
	Pan7A1	NED		77
	Pan1A2	PET		68
3	Pan1C2	6FAM	60	55
	Pan7C2	VIC		55
	Pan1C1	NED		33
	Pan4D1	PET		31

*The amplification success rate was calculated based on the genotyping of the 89 Amur leopard samples.

**Table 2:** Genetic diversity indices of the surveyed Amur leopard population (14 microsatellite markers and 24 individuals).

Locus	N	A	A_E_	PIC	H_E_	H_O_	P_ID_/loc.	P_ID-sib_/loc.	Size range	Success (%)	ADO	FA
Pan1C2*	24	4	2.84	0.577	0.66	0.54	1.74E-01	4.75E-01	164–176	90	0.025	0.058
Pan7A1*	24	3	2.66	0.549	0.64	0.75	1.95E-01	4.92E-01	169–177	96	0.058	0.029
Pan1A2*	24	4	1.91	0.428	0.49	0.46	2.85E-01	5.92E-01	179–191	95	0.094	0.027
Pan1C1*	24	3	2.03	0.428	0.52	0.50	2.97E-01	5.77E-01	164–170	62	0.126	0.038
Pan2A1*	22	3	1.81	0.366	0.46	0.46	3.62E-01	6.23E-01	223–238	88	0.038	0.066
Pan7C2*	23	2	1.52	0.282	0.35	0.26	4.63E-01	7.03E-01	200–203	86	0.222	0.061
Pan3C2*	24	2	1.44	0.258	0.31	0.38	4.98E-01	7.30E-01	104–106	100	0.102	0.006
Pan4A2*	24	2	1.44	0.258	0.31	0.29	4.98E-01	7.30E-01	138–146	96	0.052	0.008
Pan5A1*	23	2	1.40	0.246	0.29	0.35	5.17E-01	7.44E-01	192–195	96	0.017	0.017
Pan4D1*	24	3	1.41	0.272	0.30	0.21	4.80E-01	7.34E-01	161–175	64	0.144	0.030
Pan16C2*	24	2	1.23	0.169	0.19	0.13	6.49E-01	8.26E-01	179–182	91	0.067	0.031
Pan14C2*	23	2	1.19	0.146	0.16	0.09	6.93E-01	8.51E-01	202–206	61	0	0.004
Pan5D1	24	2	1.18	0.141	0.16	0.17	7.03E-01	8.56E-01	143–145	88	0	0.093
Pan2C1	24	2	1.04	0.040	0.04	0.04	9.12E-01	9.60E-01	84–86	64	0	0
Average	23.6	2.57	1.65	0.297	0.35	0.33	1.32 x 10^−5^^†^	7.16 x 10^−3^^⁋^		84	0.068	0.033

Loci are ranked from low to high values of PID-sib/loc. The amplification success rate and genotyping error rates for ADO and FA were calculated based on the 51 samples which were used for individual identification.

* 12 markers used for individual identification. Pan5D1 and Pan2C1 were used only for genetic diversity analysis.

^†^ resultant probability of identity for unrelated individuals (12 markers)

^⁋^ resultant probability of identity for siblings (12 markers)

Abbreviations include: N, sample size; A, observed number of alleles; A_E_, number of effective alleles; PIC, polymorphic information content; H_E_, expected heterozygosity; H_O_, observed heterozygosity; P_ID_/loc., probability of identity per locus; P_ID-sib_/loc., probability of identity for siblings per locus; ADO, allelic dropout; FA, false allele

### Individual identification and genetic diversity

In total, 24 individual Amur leopards were identified, which included 18 males and 6 females (Fig 1). Thirteen individuals were captured once and the others were captured 2 to 10 times (S5 Table). The expected heterozygosity ranged from 0.04 to 0.66 with an average of 0.35, indicating a low level of genetic diversity. The mean observed heterozygosity was 0.33, slightly lower than the expected heterozygosity and ranged from 0.04 to 0.75 (Table 2). The expected heterozygosity in previous studies of 0.45 reported by Rozhnov et al. [43] and 0.43 reported by Sugimoto et al. [37] were also low, indicating the genetic vulnerability of the Amur leopard population. The result of the Wilcoxon test revealed no significant sign of a recent bottleneck (probability of one tail for H excess = 0.29150), although the Garza–Williamson index was less than 0.68 (mean = 0.4205 ± 0.17018), which implies a historic bottleneck event in the population [35].

Sequencing of three mtDNA regions revealed two haplotypes with a point mutation. Two haplotypes differed at one variable site with A/T transversion at position 15596 (Table 3) in the part of cytochrome *b* [44] and some samples showed both A and T peaks, suggesting the heteroplasmy as mentioned by Sugimoto et al. [37] (Type A, n = 6; Type T, n = 14; heteroplasmy, n = 3). We did not report Amur leopard specific ORI 1, ORI 2 and KOR 1 haplotypes described by Uphyrkina et al. [38] and Hyun et al [12]. Consistent with the results of previous studies, the haplotype diversity of the population was very low.

**Table 3:** Mitochondrial DNA sequence variations in Amur leopard.

Gene	*NADH-5*				Cyt *b*	Control region		
Position*	12820	13080	13204	13206	15596	16587	16827	
Haplotype (Accession no.)								
Mitochondrion complete genome (KX655614)	A	T	G	T	T	C	A	Direct submission
Hap1	-	-	-	-	A	T	-	This study
Hap2	-	-	-	-	-	T	-	This study
TypeA (AB817078)					A			Sugimoto et al. (2014)
TypeT (AB817079)					-			Sugimoto et al. (2014)
ORI1 (AY035260, AY035227)	-	C	A	C			G	Uphyrkina et al. (2001)
ORI2 (AY035261, AY035228)	-	-	-	-			G	Uphyrkina et al. (2001)
KOR1 (MK114159, MK114160)	G	-	-	-			-	Hyun et al. (2021)

The part without information was left blank.

* Nucleotide positions correspond to the complete reference *Felis catus* mtDNA sequence Lopez, Cevario (44)

### Sample quality and amplification success

**Fig 2:**
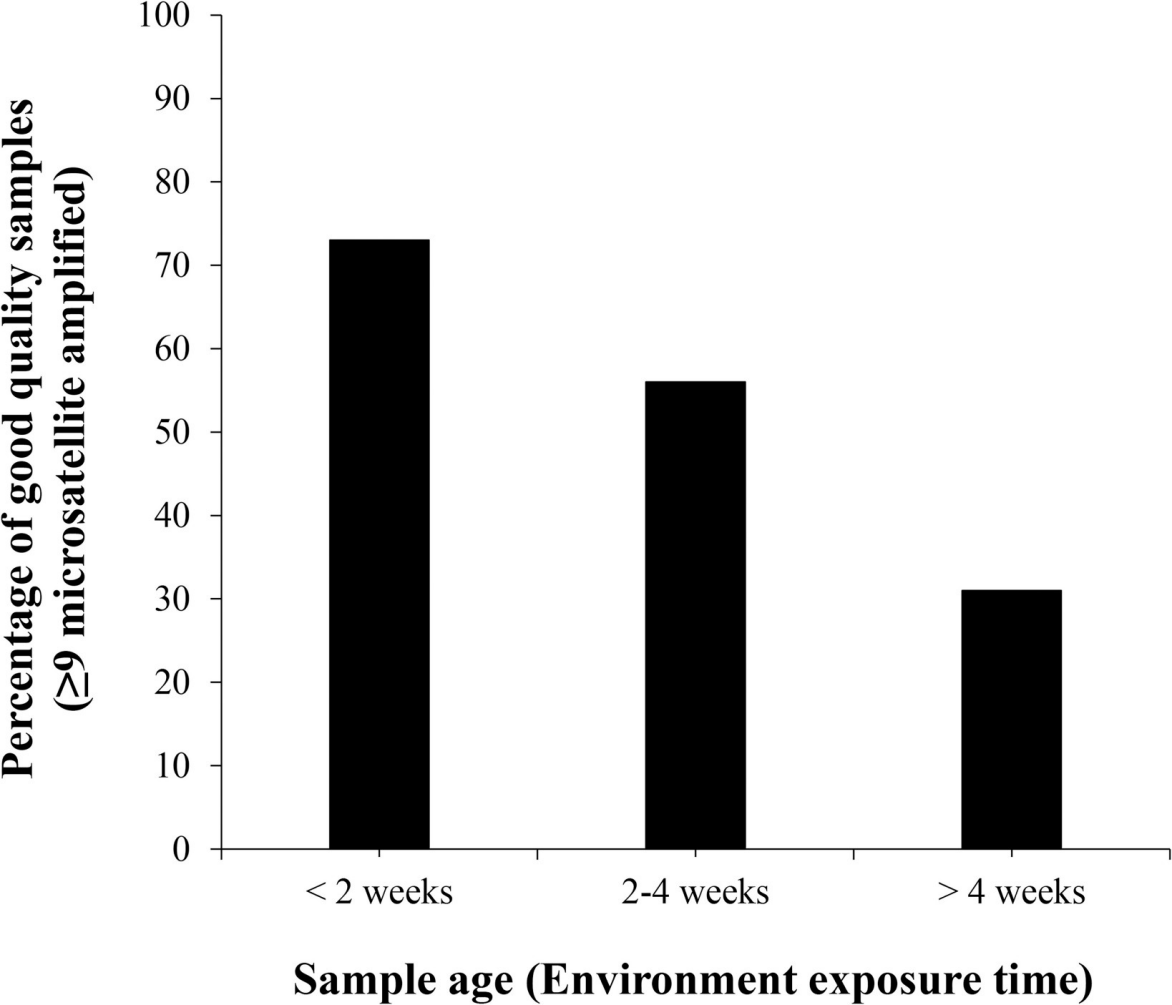
Relationship between sample age and genotyping success. Percentage of good quality samples in each sample age category.

There was a significant difference in the success rate of genotyping depending on the time from deposition to collection (*p* = 0.014, Fisher’s exact test) and no significant difference was found depending on the time from collection to DNA extraction (*p* = 0.648, Fisher’s exact test) and month of collection (*p* = 0.541, Fisher’s exact test). Since long term storage of the samples in a freezer was not problematic and there was no difference in the month of collection, obtaining fresh feces should be considered first for efficient genetic monitoring. The proportion of samples categorized as good quality were 73% in <2 weeks old samples, 56% in 2–4 weeks old samples, and 31% in >4 weeks old samples, indicating a notable decrease in quality with prolonged exposure to the environment (Fig 2). Samples collected within 2 weeks from the time of deposition accounted for 59% of all samples used for individual identification, while those collected from 2 to 4 weeks and more than 4 weeks accounted for 18% each. There was no information about the age of three fecal samples used for individual identification at the time of collection.

## Discussion

Microsatellite based DNA fingerprinting for individual identification, population estimation and to understand population genetic diversity using DNA extracted from non-invasive samples has been widely applied for several endangered species, including the Amur leopard. Previous studies of the Amur leopard focused on the cross-species amplification potential of microsatellite markers developed for the domestic cat [13, 37, 43]. Microsatellite cross-species amplification is old tested techniques that include choice advantage during marker pre-screening and cost effectiveness by avoiding marker development process. However, incidents of null allele and false allele are theoretically more with cross-species markers compared to the species-specific markers as primer binding regions may never remain conserved in the species other than the species for which markers were developed. The errors due to mis-priming are even more with non-invasive genetics as DNA yield and quality remain low. Here we applied genus specific genomic microsatellites developed for big cat species [24]. These primers were designed by analysing the whole genome sequences of four big cat species, including Amur leopard. Thus, these markers have conserved primer binding regions in Amur leopard to ensure excellent data quality and reliability.

Using the fecal DNA and a panel of 12 microsatellites amplified in three multiplex PCR reactions, we successfully identified 24 unique Amur leopard individuals, which include 18 males and 6 females. These represent approximately a quarter of the Amur leopard population of the Land of the Leopard [21, 45]. The greater number of males was in contrast to the findings of a camera trap study conducted in the Southwest of Primorsky Krai, which reported a male to female ratio of 1:1.2 [46]. The male biased sample collection was also reported in a fecal survey [37], and a camera trap survey [47]. Increased proportion of males in our sampling may have resulted either due to sampling bias or we encountered more non-resident males. However, the chances of later are grim as the Land of the Leopard represent the last surviving wild Amur leopard population in Russia. Though there exist few Amur leopards in adjacent forests in China, these are also dispersed individuals of the Land of the Leopard. Moreover, we did not report signs of population sub structuring (S1 Fig) or migrants indicating all captured individuals were likely residents. Biased or skewed sampling may likely the cause of the observed skewed sex ratio. There are more resident leopards (n~ 100) than tigers (n~ 30) in the Land of the Leopard [45]. However, our samples include more tiger feces than leopard. In the Land of the Leopard, tiger and leopard co-inhabit the same region but occupy different niches (tigers around valleys and leopards around mountain cliffs). The fecal samples were opportunistically collected by the rangers during the snow tracking surveys along the major forest roads and the valley. Both tiger and leopard are solitary and territorial animals that protects its territory through marking i.e., feces deposition. The male felids have large home ranges, encompassing the territories of several females. Therefore, male leopards are expected to deposit more feces in far places. In other noninvasive genetic studies and camera trap studies of big cat species, the proportion of males was greater than that of females [41, 48]. Therefore, collection of fecal samples with coverage of mountain ridge forest tracts during feces collection is recommended for future surveys.

Comparisons of genetic diversity are ideally conducted with the use of the same set of molecular markers. Estimation of genetic diversity with the use of microsatellites involves assessment of allelic diversity (number of alleles) and percentage of heterozygosity within the genome. Allelic diversity is marker dependent whereas heterozygosity represents the state of a population rather than the markers used. Uphyrkina et al. [13] first reported the genetic diversity (H_O_ = 0.4) of Amur leopards of Russia Far East based on 25 microsatellites and 7 individuals captured between 1993–96. Sugimoto et al. [37] studied the leopard population of Southwest Primorsky using the noninvasive genetics (sampling period: 2000–08, 13 microsatellites, and 32 individuals) and reported 43% observed heterozygosity. Rozhnov et al. [43] also studied leopards of Southwest Primorsky noninvasively (sampling period: 2010–12, 12 microsatellites, and 23 individuals) and found slightly lower observed heterozygosity (Ho– 0.39). In this study, we assessed genetic diversity of the leopards of Southwest Primorsky using 14 microsatellites and 24 individuals identified from the fecal samples collected between 2014–19. We reported 33% observed heterozygosity. These observations indicate the declining heterozygosity trend in the surviving population of Amur leopard. Inbreeding may likely be the cause of the reduction in heterozygosity as the population is small and revived from a few founders in a short time span. However, the result presented here must be considered indicative and need further validation as all the compared studies have used different microsatellite marker sets. Although samples analyzed were collected between 2014 to 2019 and thus provide an opportunity to estimate Amur leopard genetic diversity over years, the limited sample number restricts us to perform such analysis. We recommend continued population monitoring of wild Amur leopards via systematic scat sampling and using the proposed genetic methodology to precisely document genetic diversity increment or decline over time.

The haplotype diversity of the Amur leopard population is relatively small as compared to other leopard subspecies. Uphyrkina et al. [38] did leopard phylogeny and reported two Amur leopard haplotypes (ORI1 (8.3%) and ORI2 (91.7%)), which included the parts of *NADH-5* and control region. Sugimoto et al. [37] reported two haplotypes (Type A and Type T) based on part of cytochrome *b* and the ratio of Type T was about three-fold greater than that of Type A in the Amur leopard population of the Land of the Leopard. We amplified partial fragments of the cytochrome *b*, *NADH-5* and control region and reported two haplotypes that differ by a point mutation in cytochrome *b*. The observed point mutation in the cytochrome *b* represent the types A and T reported earlier by Sugimoto et al. [37]. However, we did not report previously described Amur leopard haplotypes ORI1 and ORI2. The loss of maternal lineage further could be indicative of the poor genetic health and inbreeding in the wild Amur leopards.

The Amur leopard population was reduced to about 20 individuals in the late 1900s due to rampant hunting and habitat destruction [49]. Thus, a recent genetic bottleneck footprint was expected in the surviving population of Amur leopard. However, no significant recent bottleneck was detected in the results of the BOTTLENECK program. The BOTTLENECK program compares H_E_ and H_EQ_ (heterozygosity expected at mutation-drift equilibrium) values to find excessive H_E_, and find the evidence of a relatively recent bottleneck occurred between generations 0.2–4Ne (bottleneck effective size) [34, 50]. A recent bottleneck could go undetected due to sampling bias, insufficient loci, short species history, or subpopulation structure [50–52]. Sampling bias may likely be the cause as we tested the sufficiently large number of microsatellite loci and we observed no population sub structuring (S1 Fig) in the analyzed individuals. Fecal samples were opportunistically collected and identified male individuals outnumbered the female. The Land of the Leopard has about 100 Amur leopards and we sampled only 24 individuals. Thus, the analyzed samples were not representative of the entire population. Therefore, further studies with larger sample sizes are needed [53, 54]. On the other hand, a historical bottleneck was detected using the Garza–Williamson M ratio, which is the ratio between the number of alleles and the range in allele size. Since recovery of the number of alleles is very slow, traces of relatively old bottlenecks, occurring more than 100–1000 generations ago can be found [35]. This suggests that the Amur leopard population might have decreased before the recent population decline.

Monitoring individuals with genetic tagging has many advantages. It is possible to identify demographic structures and to keep up with population trends, such as abundance and birth rates and death rates, using noninvasive samples [55]. Also, the use of genetic tags is an effective strategy to monitor wide ranging species that cross borders [56, 57]. Genetic tagging of Amur leopard can help cooperate Russia and China since some border population individuals are now found in both sides [58]. It would also be useful to identify individuals that cause conflicts with humans or victims by poaching [59, 60]. Therefore, we suggest genetic monitoring using above 12 markers which is practical and cost effective since it only requires three multiplex PCR and it has advantage in species-specific character [55]. The cost of genotyping can be greatly reduced because 12 markers can be analyzed by three PCR amplifications of four markers each. Comparatively, in a previous study Sugimoto et al. [37], six multiplex PCR reactions were conducted to amplify 13 markers, indicating that the former required twice as many PCR reactions. A cost comparison with the study conducted by Rozhnov et al. [43] was not possible due to lack of methodological details. Nonetheless, considering the dye label, marker amplicon size, and instrument genetic analyzer reading efficiency, their genotyping efforts included more than three attempts per sample, which is more expensive than the proposed methodology.

An estimation of the population was not conducted because the samples were obtained opportunistically, but a systematic design of fecal sample collection would even allow reliable population estimation using the capture recapture method [61]. In particular, the use of fresh samples with high genotyping success rates in large numbers may yield better results in the analysis. Through extended monitoring with sufficient samples in a wide range, finding out population trends of Amur leopards and planning future conservation strategies will be possible [62, 63]. Noninvasive genetic analysis can aid traditional snow tracking survey and camera trap survey analysis for robust population estimation and even combining individual information of methods will be possible [64–66].

## Supporting information

S1 Fig Genetic structure in the Amur leopard population of the Land of the Leopard, (Primorsky Krai, Russia) using STRUCTURE 2.3.4 [67] (8 iterations of 10000 burn-in followed by 10000 MCMC steps from K = 1 to K = 10). (A) Log posterior probability of 14 microsatellites data for K number of clusters visualized by STRUCTURE HARVESTER web 0.6.94 [68]. (B) Bar plot at K = 1 and (C) Bar plot at K = 2.
(TIF)

S1 Table Details of novel primer designed to amplify partial fragment of the mitochondrial control region. (DOCX)

S2 Table Categorical tables–sampling factors and sample quality. (DOCX)

S3 Table 89 leopard scat samples information. (DOCX)

S4 Table Results of microsatellite marker prescreening using 35 randomly selected leopard samples. (DOCX)

S5 Table Results of individual identification for the fecal samples collected during the six winters. (DOCX)
