# Supplementary figures and images for: Efficient and cost-effective non-invasive population monitoring as a method to assess the genetic diversity of the last remaining population of Amur leopard (Panthera pardus orientalis) in the Russia Far East
Source: PLoS One. 2022 Jul 6;17(7):e0270217. doi: 10.1371/journal.pone.0270217 (PMC9258825; doi:10.1371/journal.pone.0270217)

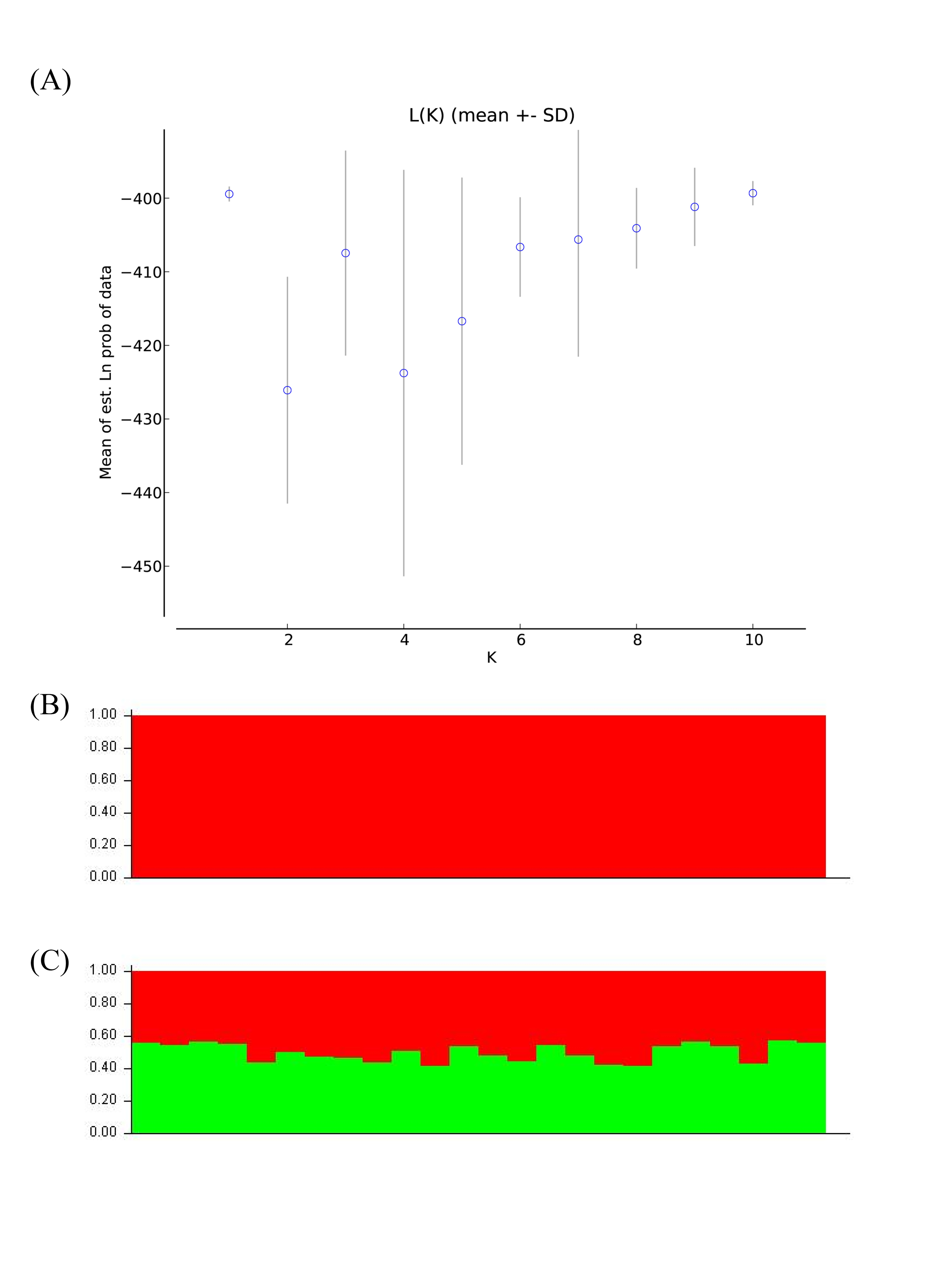

Supplement: S1 Fig — (A) Log posterior probability of 14 microsatellites data for K number of clusters visualized by STRUCTURE HARVESTER web 0.6.94 [68]. (B) Bar plot at K = 1 and (C) Bar plot at K = 2. (TIF) [file pone.0270217.s001.tif]
