# Supplementary material for: Efficient and cost-effective non-invasive population monitoring as a method to assess the genetic diversity of the last remaining population of Amur leopard (Panthera pardus orientalis) in the Russia Far East
Source: PLoS One. 2022 Jul 6;17(7):e0270217. doi: 10.1371/journal.pone.0270217 (PMC9258825; doi:10.1371/journal.pone.0270217)
Supplement: S1 Table — (DOCX) [file pone.0270217.s002.docx]

**S1 Table. Details of novel primer designed to amplify partial fragment of the mitochondrial control region**

| Target region | Primer | Sequence | Reference |
| --- | --- | --- | --- |
| control region | PC1 | CCCCCGGGTGAAACACTAAT | This study |
|  | PC2 | CCTGAAGTAAGAACCAGATG |  |

PCR amplification conditions: Each 30-μL PCR reaction mixture consisted of 1× PCR buffer with 3 mM MgCl_2_, 0.2 mM of each dNTP, 3 μg of BSA, 0.7 μM of each primer, 1.5 U of i-StarTaq™ polymerase, and 3 μL of template DNA. PCR cycling was conducted using a Takara PCR Thermal Cycler Dice system. The PCR conditions for amplification of the control region consisted of an initial denaturation step at 94°C for 5 min, followed by 40 cycles of denaturation at 94°C for 30 s, annealing at 55°C for 1 min, and extension at 72°C for 1 min, and a final extension step at 72°C for 10 min.
