# Supplementary material for: Efficient and cost-effective non-invasive population monitoring as a method to assess the genetic diversity of the last remaining population of Amur leopard (Panthera pardus orientalis) in the Russia Far East
Source: PLoS One. 2022 Jul 6;17(7):e0270217. doi: 10.1371/journal.pone.0270217 (PMC9258825; doi:10.1371/journal.pone.0270217)
Supplement: S2 Table — (DOCX) [file pone.0270217.s003.docx]

**S2 Table. Categorical tables – sampling factors and sample quality**

| Sample quality  Sample age | Good  (≥ 9 loci amplified) | Medium  (8 to 5 loci amplified) | Poor  (≤ 4 loci amplified) | Total |
| --- | --- | --- | --- | --- |
| <2 weeks | 30 | 4 | 7 | 41 |
| 2–4 weeks | 9 | 2 | 5 | 16 |
| >4 weeks | 9 | 5 | 14 | 28 |
| Total | 48 | 11 | 26 | **85^*^** |

1. Sample quality and sample age (time from deposition to collection; environment exposure time).

* 4 samples had no information about approximate age when collected.

(b) Sample quality and sample storage (time from collection to DNA extraction).

| Sample quality      Sample storage | Good  (≥ 9 loci amplified) | Medium  (8 to 5 loci amplified) | Poor  (≤ 4 loci amplified) | Total |
| --- | --- | --- | --- | --- |
| ≤ 2 years | 28 | 6 | 12 | 46 |
| 2–4 years | 11 | 2 | 10 | 23 |
| ≥ 4 years | 11 | 3 | 5 | 19 |
| total | 50 | 11 | 27 | **88^*^** |

* 1 sample had no information about collection date.

(c) Sample quality and sample collection month.

| Sample quality      Collection month | Good  (≥ 9 loci amplified) | Medium  (8 to 5 loci amplified) | Poor  (≤ 4 loci amplified) | Total |
| --- | --- | --- | --- | --- |
| Nov–Dec | 10 | 3 | 6 | 19 |
| Jan | 15 | 1 | 5 | 21 |
| Feb | 12 | 3 | 4 | 19 |
| Mar–Apr | 13 | 4 | 12 | 29 |
| total | 50 | 11 | 27 | **88^*^** |

* 1 sample had no information about month of collection.
