# Supplementary material for: Efficient and cost-effective non-invasive population monitoring as a method to assess the genetic diversity of the last remaining population of Amur leopard (Panthera pardus orientalis) in the Russia Far East
Source: PLoS One. 2022 Jul 6;17(7):e0270217. doi: 10.1371/journal.pone.0270217 (PMC9258825; doi:10.1371/journal.pone.0270217)
Supplement: S3 Table — (DOCX) [file pone.0270217.s004.docx]

**S3 Table. 89 leopard scat samples information**

| Sample ID (CGRB) | Collection date | Latitude X | Longitude Y | Estimated sample age at the time of collection | Sampling site | Species ID (Field biologist) | Species ID (genetic method) | Loci genotyped successfully |
| --- | --- | --- | --- | --- | --- | --- | --- | --- |
| 743 | 2014-03-14 | 43.53349 | 131.72275 | ≥1 month | animal trail | PPO | PPO | 10 |
| 744 | 2014-03-17 | 43.50243 | 131.64404 | ≥1 month | animal trail | PPO | PPO | 1 |
| 746 | 2014-03-18 | 43.46303 | 131.56487 | ≥1 month | animal trail | PPO | PPO | 9 |
| 833 | 2014-03-13 | 43.40091 | 131.64737 | ≥1 month | animal trail | PPO | PPO | 12 |
| 866 | 2014-12-05 | 43.08017 | 131.55151 | 2-3 days | off road | PPO | PPO | 12 |
| 884 | 2015-01-05 | 43.47801 | 131.68324 | ≥7 days | animal trail | PPO | PPO | 12 |
| 898 | 2015-01-08 | 43.44524 | 131.60172 | ≤7 days | off road | PPO | PPO | 4 |
| 909 | 2015-01-13 | 43.64516 | 131.73055 | ≥2 weeks | animal trail | PPO | PPO | 9 |
| 928 | 2015-01-22 | 43.65046 | 131.74496 | ≥2 weeks | animal trail | PPO | PPO | 12 |
| 954 | 2015-02-01 | 43.47768 | 131.63580 | ≥7 days | animal trail | PTA | PPO | 12 |
| 987 | 2015-02-20 | 43.08327 | 131.40457 | ≥2 weeks | animal trail | PPO | PPO | 6 |
| 988 | 2015-02-20 | 43.08327 | 131.40457 | ≥2 weeks | animal trail | PPO | PPO | 12 |
| 1007 | 2015-03-16 | 42.90556 | 131.21800 | 24 h | animal trail | PPO | PPO | 10 |
| 1022 | 2015-03-19 | 43.40481 | 131.33992 | ≥1 month | animal trail | PPO | PPO | 0 |
| 1027 | 2015-03-19 | 43.36179 | 131.38844 | ≤7 days | animal trail | PPO | PPO | 12 |
| 1032 | 2015-03-21 | 42.84064 | 131.19002 | ≥1 month | road | PPO | PPO | 1 |
| 1044 | 2015-03-25 | 42.83907 | 130.93774 | ≥1 month | animal trail | unknown | PPO | 5 |
| 1047 | 2015-03-25 | 42.83277 | 130.98409 | ≥7 days | animal trail | unknown | PPO | 5 |
| 1049 | 2015-03-25 | 42.83318 | 130.98448 | ≥1 month | animal trail | PPO | PPO | 2 |
| 1077 | 2015-12-18 | 43.09628 | 131.54436 | ≥1 month | animal trail | PPO | PPO | 2 |
| 1078 | 2015-12-16 | 43.22337 | 131.49266 | ≤7 days | road | unknown | PPO | 12 |
| 1082 | 2016-02-02 | 43.36917 | 131.53262 | ≤7 days | off road | PPO | PPO | 12 |
| 1098 | 2016-02-27 | 43.15721 | 131.37334 | ≥2 weeks | animal trail | unknown | PPO | 9 |
| 1099 | 2016-02-16 | 43.42979 | 131.50655 | 24 h | off road | PPO | PPO | 12 |
| 1100 | 2016-02-16 | 43.42830 | 131.51227 | 24 h | off road | PPO | PPO | 9 |
| 1101 | 2016-02-17 | 43.43389 | 131.50195 | ≥2 weeks | animal trail | unknown | PPO | 1 |
| 1104 | 2016-03-09 | 43.48265 | 131.54492 | unknown | animal trail | PPO | PPO | 12 |
| 1106 | 2016-03-10 | 43.47611 | 131.68185 | ≥1 month | animal trail | PPO | PPO | 0 |
| 1108 | 2016-03-18 | 43.09834 | 131.55350 | ≥3 months | human trail | PTA | PPO | 4 |
| 1109 | 2016-03-18 | 43.10830 | 131.53476 | ≥3 months | human trail | PPO | PPO | 1 |
| 1111 | 2016-03-17 | 43.43125 | 131.65019 | ≥3 months | animal trail | PPO | PPO | 7 |
| 1184 | 2016-12-29 | 43.54441 | 131.79678 | ≤7 days | animal trail | PPO | PPO | 7 |
| 1185 | 2016-12-29 | 43.54317 | 131.78233 | ≤7 days | animal trail | PPO | PPO | 12 |
| 1208 | 2017-01-11 | 43.36197 | 131.67128 | 2-3 days | animal trail | PPO | PPO | 11 |
| 1213 | 2017-01-31 | 43.62043 | 131.43069 | ≥1 month | animal trail | PPO | PPO | 12 |
| 1236 | 2017-03-01 | 43.26347 | 131.47633 | 24 h | off road | PPO | PPO | 12 |
| 1246 | 2017-03-28 | 43.10831 | 131.53429 | unknown | unknown | PPO | PPO | 1 |
| 1247 | 2017-01-22 | 43.50110 | 131.53105 | unknown | unknown | unknown | PPO | 9 |
| 1250 | 2017-03-28 | 43.06659 | 131.31522 | ≥1 month | animal trail | unknown | PPO | 1 |
| 1264 | 2017-03-28 | 43.06495 | 131.32069 | ≥2 weeks | animal trail | PPO | PPO | 0 |
| 1266 | 2017-04-04 | 43.10591 | 131.55220 | ≥1 month | unknown | PPO | PPO | 0 |
| 1298 | 2017-02-13 | 43.35833 | 131.59975 | ≥1 month | road | PPO | PPO | 1 |
| 1304 | 2017-12-16 | 43.10011 | 131.57597 | 2-3 days | off road | PPO | PPO | 9 |
| 1306 | 2017-12-19 | 43.10030 | 131.57039 | ≤7 days | off road | PPO | PPO | 8 |
| 1311 | 2017-11-23 | 43.01942 | 131.31120 | ≤7 days | off road | PPO | PPO | 3 |
| 1314 | 2017-12-16 | 43.10089 | 131.57230 | 24 h | off road | PPO | PPO | 9 |
| 1315 | 2017-11-07 | 43.08330 | 131.40485 | old | animal trail | PPO | PPO | 5 |
| 1323 | 2018-01-07 | 43.45673 | 131.60611 | ≤7 days | animal trail | PPO | PPO | 12 |
| 1324 | 2018-01-11 | 43.10596 | 131.55023 | ≤7 days | off road | PPO | PPO | 12 |
| 1327 | 2018-01-16 | 43.35068 | 131.46916 | old | animal trail | PPO | PPO | 5 |
| 1330 | 2018-01-24 | 43.66786 | 131.77203 | ≤7 days | animal trail | PPO | PPO | 9 |
| 1332 | 2018-01-26 | 43.35077 | 131.66730 | ≥1 month | animal trail | unknown | PPO | 12 |
| 1336 | 2018-01-30 | 43.30462 | 131.67389 | ≥1 month | animal trail | PPO | PPO | 12 |
| 1337 | 2018-01-30 | 43.30044 | 131.67464 | ≥1 month | animal trail | PPO | PPO | 9 |
| 1338 | 2018-01-30 | 43.30027 | 131.67464 | ≥3 months | animal trail | PPO | PPO | 2 |
| 1340 | 2018-02-10 | 43.10104 | 131.55815 | ≥7 days | animal trail | PPO | PPO | 9 |
| 1350 | 2017-11-23 | 43.01945 | 131.31120 | ≤7 days | off road | PPO | PPO | 12 |
| 1352 | 2018-03-04 | 43.35023 | 131.46953 | ≥1 month | animal trail | PPO | PPO | 6 |
| 1358 | 2018-11-22 | 43.23735 | 131.64250 | 1week | animal trail | PPO | PPO | 1 |
| 1367 | 2018-12-06 | 42.87117 | 131.15068 | 2 week | animal trail | PPO | PPO | 11 |
| 1371 | 2018-12-11 | 42.55182 | 130.60477 | 1-2 week | animal trail | PPO | PPO | 2 |
| 1372 | 2018-12-11 | 42.54487 | 130.60898 | 2-3 week | animal trail | PPO | PPO | 12 |
| 1373 | 2018-12-11 | 42.54487 | 130.60898 | 2 week | animal trail | PPO | PPO | 2 |
| 1374 | 2018-12-11 | 42.54487 | 130.60898 | 3-4 week | animal trail | PPO | PPO | 11 |
| 1385 | 2018-12-20 | 42.92488 | 131.16266 | 4 days | animal trail | PPO | PPO | 12 |
| 1386 | 2018-12-20 | 42.94702 | 131.16208 | 1 week | animal trail | PPO | PPO | 2 |
| 1395 | 2019-01-16 | 43.34965 | 131.46967 | 4 weeks | animal trail | PPO | PPO | 9 |
| 1403 | 2019-01-19 | 43.31257 | 131.72737 | 2 days | off road | PPO | PPO | 12 |
| 1404 | 2019-01-20 | 43.43127 | 131.65016 | >8 weeks | animal trail | PPO | PPO | 1 |
| 1407 | 2019-01-29 | 43.35241 | 131.37888 | 4-5 days | animal trail | PPO | PPO | 4 |
| 1408 | 2019-01-29 | 43.35241 | 131.37888 | 2-3 days | animal trail | PPO | PPO | 12 |
| 1412 | 2019-01-27 | 43.62501 | 131.48897 | >4 weeks | animal trail | PPO | PPO | 4 |
| 1419 | 2019-02-06 | 43.11105 | 131.52534 | <4 weeks | human trail | unknown | PPO | 7 |
| 1430 | 2019-02-12 | 43.05507 | 131.27173 | 3 weeks | animal trail | PPO | PPO | 11 |
| 1441 | 2019-02-11 | 43.31956 | 131.72565 | 24 hours | off road | PPO | PPO | 12 |
| 1442 | 2019-02-11 | 43.31956 | 131.72565 | 24 hours | off road | PPO | PPO | 6 |
| 1443 | 2019-02-20 | 43.18522 | 131.38351 | ≤7 days | animal trail | PPO | PPO | 12 |
| 1446 | 2019-02-27 | 43.10831 | 131.53499 | ≤1 month | human trail | PPO | PPO | 4 |
| 1447 | 2019-02-27 | 43.09840 | 131.55360 | ≥1 month | human trail | PPO | PPO | 12 |
| 1456 | 2019-02-25 | 43.28191 | 131.52156 | ≥2 weeks | animal trail | PPO | PPO | 0 |
| 1463 | 2019-02-25 | 43.27851 | 131.51680 | ≥7 days | animal trail | PPO | PPO | 12 |
| 1464 | 2019-03-04 | 43.11170 | 131.52524 | ≥2 weeks | road | PPO | PPO | 9 |
| 1465 | 2019-03-04 | 43.10832 | 131.53447 | 1 day | road | PPO | PPO | 1 |
| 1466 | 2019-03-06 | 43.10252 | 131.54312 | 1-2 weeks | road | PPO | PPO | 9 |
| 1468 | 2019-03-15 | 43.24387 | 131.63670 | 3-7 days | animal trail | PPO | PPO | 9 |
| 1473 | 2019-03-20 | 43.37733 | 131.56012 | 1-2 weeks | animal trail | PPO | PPO | 9 |
| 1474 | 2019-03-20 | 43.37538 | 131.56641 | 1-2 weeks | animal trail | PPO | PPO | 12 |
| 1480 | 2019-04-22 | 43.31707 | 131.72108 | 1-2days | off rood | PPO | PPO | 12 |
| 1487 | unknown | unknown | unknown | unknown | unknown | unknown | PPO | 12 |

Abbreviations include: PPO, *Panthera pardus orientalis*; PTA, *Panthera tigris altaica*
