# Supplementary material for: Efficient and cost-effective non-invasive population monitoring as a method to assess the genetic diversity of the last remaining population of Amur leopard (Panthera pardus orientalis) in the Russia Far East
Source: PLoS One. 2022 Jul 6;17(7):e0270217. doi: 10.1371/journal.pone.0270217 (PMC9258825; doi:10.1371/journal.pone.0270217)
Supplement: S4 Table — (DOCX) [file pone.0270217.s005.docx]

| **Locus** | **A** | **ADO** | **FA** | **Amplification success rate** | **P_ID_/loc.** | **P_ID-sib_/loc.** |
| --- | --- | --- | --- | --- | --- | --- |
| Pan1C2 | 4 | 0.038 | 0.079 | 94% | 1.65.E-01 | 4.66.E-01 |
| Pan 7A1 | 3 | 0.076 | 0.050 | 95% | 2.17.E-01 | 5.01.E-01 |
| Pan 1A2 | 4 | 0.086 | 0.044 | 93% | 2.74.E-01 | 5.67.E-01 |
| Pan 1C1 | 3 | 0.134 | 0.067 | 87% | 3.29.E-01 | 6.03.E-01 |
| Pan 7C2 | 2 | 0.186 | 0.083 | 88% | 4.05.E-01 | 6.41.E-01 |
| Pan 2A1 | 4 | 0.046 | 0.072 | 89% | 3.89.E-01 | 6.56.E-01 |
| Pan 4A2 | 2 | 0.000 | 0.010 | 97% | 4.93.E-01 | 7.18.E-01 |
| Pan 4D1 | 3 | 0.163 | 0.047 | 99% | 4.96.E-01 | 7.33.E-01 |
| Pan 6A1 | 3 | 0.250 | 0.147 | 94% | 5.22.E-01 | 7.50.E-01 |
| Pan 5A1 | 2 | 0.000 | 0.025 | 96% | 5.39.E-01 | 7.51.E-01 |
| Pan 3C2 | 2 | 0.082 | 0.008 | 100% | 5.54.E-01 | 7.61.E-01 |
| Pan 16C2 | 2 | 0.133 | 0.029 | 91% | 7.45.E-01 | 8.74.E-01 |
| Pan 14C2 | 2 | 0.000 | 0.006 | 91% | 7.83.E-01 | 8.94.E-01 |
| Pan 5D1 | 2 | 0.000 | 0.097 | 91% | 7.88.E-01 | 8.97.E-01 |
| Pan 2C1 | 2 | 0.000 | 0.000 | 100% | 9.41.E-01 | 9.72.E-01 |
| Pan 1A1 | 1 | 0.000 | 0.006 | 81% | 1 | 1 |
| Pan 4A1 | 1 | 0.000 | 0.011 | 91% | 1 | 1 |
| Pan 8C2 | 1 | 0.000 | 0.000 | 96% | 1 | 1 |
| Pan 3D2 | 1 | 0.000 | 0.013 | 91% | 1 | 1 |
| Pan 6A2 | 1 | 0.000 | 0.000 | 74% | 1 | 1 |
| Pan 6C2 | 1 | 0.000 | 0.000 | 84% | 1 | 1 |
| Pan 2D1 | 1 | 0.000 | 0.000 | 81% | 1 | 1 |
| Pan 3D1 | 1 | 0.000 | 0.000 | 69% | 1 | 1 |
| Pan 1D2 | 1 | 0.000 | 0.000 | 92% | 1 | 1 |
| Pan 3A2 | 1 | 0.000 | 0.006 | 91% | 1 | 1 |
| Pan 9C2 | 1 | 0.000 | 0.000 | 88% | 1 | 1 |
| Pan 1D1 | 1 | 0.000 | 0.000 | 87% | 1 | 1 |
| Pan 15C2 | 1 | 0.000 | 0.007 | 90% | 1 | 1 |
| Pan 3A1 | 1 | 0.000 | 0.000 | 92% | 1 | 1 |
| Pan 2D2 | 1 | 0.000 | 0.005 | 100% | 1 | 1 |
| Pan 10C2 | 1 | 0.000 | 0.000 | 99% | 1 | 1 |
| Pan 8A1 | 1 | 0.000 | 0.000 | 99% | 1 | 1 |

**S4 Table. Results of microsatellite marker prescreening using 35 randomly selected leopard samples**

Abbreviations include: A, observed number of alleles; ADO, allelic dropout; FA, false allele; P_ID_/loc., probability of identity per locus; P_ID-sib_/loc., probability of identity for siblings per locus.
