# Supplementary material for: Efficient and cost-effective non-invasive population monitoring as a method to assess the genetic diversity of the last remaining population of Amur leopard (Panthera pardus orientalis) in the Russia Far East
Source: PLoS One. 2022 Jul 6;17(7):e0270217. doi: 10.1371/journal.pone.0270217 (PMC9258825; doi:10.1371/journal.pone.0270217)
Supplement: S5 Table — (DOCX) [file pone.0270217.s006.docx]

**S5 Table. Results of individual identification for the fecal samples collected during the six winters**

| Individuals | Total no. of observations (captures) | Winters observed | | | | | | |
| --- | --- | --- | --- | --- | --- | --- | --- | --- |
|  |  | 2013-2014 | 2014-2015 | 2015-2016 | 2016-2017 | 2017-2018 | 2018-2019 | No information |
| LM1 | 3 | 1 | 1 |  | 1 |  |  |  |
| LM2 | 1 | 1 |  |  |  |  |  |  |
| LM3 | 1 |  | 1 |  |  |  |  |  |
| LM4 | 2 |  | 1 | 1 |  |  |  |  |
| LM5 | 3 |  | 2 |  |  | 1 |  |  |
| LM6 | 10 |  | 1 | 1 |  | 4 | 4 |  |
| LM7 | 2 |  | 1 |  |  |  | 1 |  |
| LM8 | 1 |  | 1 |  |  |  |  |  |
| LM9 | 3 |  |  | 2 | 1 |  |  |  |
| LM10 | 1 |  |  |  | 1 |  |  |  |
| LM11 | 1 |  |  |  | 1 |  |  |  |
| LM12 | 5 |  |  |  |  | 3 | 2 |  |
| LM13 | 2 |  |  |  |  | 1 | 1 |  |
| LM14 | 3 |  |  |  |  |  | 2 | 1 |
| LM15 | 1 |  |  |  |  |  | 1 |  |
| LM16 | 3 |  |  |  |  |  | 3 |  |
| LM17 | 1 |  |  |  |  |  | 1 |  |
| LM18 | 1 |  |  |  |  |  | 1 |  |
| LF1 | 1 | 1 |  |  |  |  |  |  |
| LF2 | 1 |  |  | 1 |  |  |  |  |
| LF3 | 1 |  |  | 1 |  |  |  |  |
| LF4 | 1 |  |  |  | 1 |  |  |  |
| LF5 | 2 |  |  |  |  |  | 2 |  |
| LF6 | 1 |  |  |  |  | 1 |  |  |
| Total | 51 | 3 | 8 | 6 | 5 | 10 | 18 | 1 |

Abbreviations include: LM, leopard male; LF, leopard female
